# The Accuracy of Noninvasive Imaging Techniques in Diagnosis of Carotid Plaque Morphology

**DOI:** 10.3889/oamjms.2015.039

**Published:** 2015-03-27

**Authors:** Detelina Valchkova Lukanova, Nadelin Krasimirov Nikolov, Kameliya Zaharieva Genova, Mario Draganov Stankev, Elisaveta Valcheva Georgieva

**Affiliations:** 1 *Clinic of Vascular Surgery and Angiology, MBAL “National Heart Hospital”, Sofia, Bulgaria*; 2 *Department of Diagnostic and Interventional Radiology, MBAL “National Heart Hospital”, Sofia, Bulgaria*

**Keywords:** carotid, noninvasive imaging, vulnerable plaque

## Abstract

**BACKGROUND:**

The stroke is leading cause of death and severe disability worldwide. Atherosclerosis is responsible for over 30% of all ischemic strokes. It has been recently discovered that plaque morphology may help predict the clinical behavior of carotid atherosclerosis and determine the risk of stroke. The noninvasive imaging techniques have been developed to evaluate the vascular wall in an attempt to identify “vulnerable plaques”.

**AIM:**

The purpose is to investigate the diagnostic accuracy of ultrasound, multidetector computed tomography and magnetic resonance imaging in the identification of plaque components associated with plaque vulnerability.

**MATERIAL AND METHODS:**

One hundred patients were admitted for carotid endarterectomy for high grade carotid stenosis. We defined the diagnostic value of B-mode ultrasound of carotid plaque in a half, and the accuracy of multidetector computed tomography and magnetic resonance imaging, in the other group, for detection of unstable carotid plaque. The reference standard was histology.

**RESULTS:**

Sensitivity of ultrasound, multidetector computed tomography and magnetic resonance imaging is 94%, 83% and 100%, and the specificity is 93%, 73% and 89% for detection of unstable carotid plaque.

**CONCLUSION:**

The ultrasound has high accuracy for diagnostics of carotid plaque morphology, magnetic resonance imaging has high potential for tissue differentiation and multidetector computed tomography determines precisely degree of stenosis and presence of ulceration and calcifications. The three noninvasive imaging modalities are complementary for optimal evaluation of the morphology of carotid plaque. This will help to determine the risk of stroke and to decide on the best treatment – carotid endarterectomy or carotid stenting.

## Introduction

Cardiovascular diseases, in particular cerebrovascular disease (CVD), are the leading cause of morbidity, mortality and severe disability in Bulgaria and worldwide. Atherosclerosis of the carotid arteries is responsible for over 30% of ischemic strokes [1-3] [4-11].

Several large randomized multicenter studies (NASCET, ECST, ACAS, ACST) showed the benefit of carotid endarterectomy (CEA), and recently other studies (CAVATAS, SAPPHIRE, CREST) and carotid artery stenting (CAS), for the prevention of stroke in both symptomatic and asymptomatic patients [4-11]. In these studies, the only criterion for patients’ selection at high risk of stroke is the degree of stenosis of the internal carotid artery (ICA) [4, 12-16]. They also demonstrated that many patients with high-grade stenosis (>70%) did not get a stroke even with medication. On the other hand, many authors indicate that most of the neurological symptoms are found in patients with carotid stenosis <70% [6, 13, 14, 17]. It is clear that other factors such as histological composition of the plaque, in addition to the degree of stenosis, are responsible for determining the risk of stroke [5, 6, 18]. This gave way to the theory of “unstable atherosclerotic plaque.” In the last decade, numerous studies (ICAROS,, ACSRS, Tromso) proved the role of carotid plaque morphology as an independent predictor of ischemic events [6, 19, 21]. Precise non-invasive imaging techniques received rapid development to study the vascular wall in an attempt to find “unstable plaque”: ultrasound, magnetic resonance tomography and multidetector computed tomography. The challenges for these research tools are to identify high-risk patients who have lesions prone to thrombosis and embolism, before the onset of cerebrovascular accident. Imaging methods must have the potential not only for screening atherosclerosis but also for helping to distinguish a stable from unstable plaque and to divide patients in low-risk and high- risk groups for cardiovascular complications. This will optimize prevention and treatment strategies in endangered population and improve the process of deciding how to treat - invasive or with drugs. Furthermore, it will help in selection of invasive therapeutic method - carotid endarterectomy (CEA) or carotid angioplasty and stenting (CAS) [22-27].

The aim of this study was to determine prospectively the diagnostic accuracy of ultrasound (US), multidetector computed tomography (MDCT) and magnetic resonance imaging (MRI) in assessing the composition and morphology of carotid plaque and to compare it with histological examination of specimen from carotid endarterectomy.

## Materials and Methods

We studied prospectively 100 consecutive patients with >60% carotid stenosis (unilateral or bilateral) of 116 atherosclerotic plaques. Of these, 75 were men and 25 women with an average age 66. Symptomatic were 32 and 68 - asymptomatic. Symptomatic patients are those with amaurosis fugax, transient ischemic attacks and hemispheric stroke within the last 6 months. We did not include patients with bilateral thrombosis of the carotid arteries and those with severe neurological deficit and also symptomatic patients with cardioembolic status based on ECG and echocardiography evidences. We excluded patients with carotid stenoses, caused by plaques type V on B-mode ultrasound image. We considered risk factors for atherosclerosis and medication, and the presence of diabetes mellitus, hypertension, coronary artery disease, peripheral artery disease and smoking. We examined total cholesterol and C-reactive protein. Patients were divided into two groups: in the first group of 50 patients (57 plaques) we determined diagnostic value of B-scan ultrasonography by visual analysis to identify the composition and morphology of the carotid plaque, while in the other 50 patients (59 plaques) we evaluated the capabilities of magnetic resonance imaging (MRI) - 25 patients (30 plaques) and of multidetector computed tomography (MDCT) - 25 patients (29 plaques) to determine the composition and morphology of atherosclerotic carotid plaque. As reference standard we used histological examination of specimen from CEA.

### Ultrasound

All 100 patients were examined. We used a duplex scanner Aloka Prosound alpha-6 with 7.4 MHz linear transducer. The carotid arteries were examined by both B-mode and color flow modalities. The degree of stenosis was evaluated by ECST criteria and analysis of the spectral curve. The visual assessment of the structure and morphology of carotid atherosclerotic plaque is done by the B-mode in gray scale with high resolution in combination with a compound-scanning in real time. For anechogenic plaques we used color flow Doppler and the power Doppler. For assessment of carotid plaques with carotid duplex sonography (CDS) three basic parameters were used: echogenicity, structure and surface of the plaque. We use Gray-Weale/Geroulacos classification: type I (homogeneous anechogenic plaque), type II (mainly anechogenic with hyperechogenic areas <50% of the area), type III (mainly echogenic with hypoechogenic sections <50% of the area), type IV (homogeneous echogenic plaque) and type V (unclassified calcium plaques with acoustic shadowing of the underlying lesion). Plaques type I and II are unstable, and type III and IV are stable. Plaques types V are excluded from the study. Surface of the plaque is defined as: smooth, rough (fissures deep 0.3-0.9 mm) or ulcers with depth >1 mm. The screening of patients and preoperative diagnostics was performed with CDS from two independent observers with inter observer Kappa, K *=* 0.96 – nearly perfect agreement.

### Multidetector computed tomography

Studies were carried out on Aquilion 64 slice CT scanner (Toshiba Medical Systems Corporation, Tokyo, Japan) with contrast enhancement – 80 ml Jodixanol (Visipaque, Amershan). We determined the degree of carotid stenosis and examined the luminal surface of plaque for ulceration or narrowing. Morphological assessment of atherosclerotic plaque comprises of: structural integrity of the plaque by measuring the density of its various components in Hounsfield units (HU), the surface of the plaque and the degree of calcium deposits. Sure Plaque program automatically calculated area and volume of the plaque and the proportion of its components: lipid-necrotic core, fibrosis q blood products and calcium. It outlines the components of atherosclerotic plaque in the region of interest of axial slices and they are identified by measuring the density in HU. Classification is in three groups: soft plaques: lipid (<50 HU); mixed plaques: fibrosis and lipids (50 -149 HU); calcium plaques (> 150 HU). We have adopted plaques with density <149 HU for risk ones (soft and mixed) and those with a density >150 HU for solid. The evaluation of the morphological type of a plaque as stable or unstable is a complex based on the surface area, volume, density, and characteristics of the surface. So the plaques detected with MDCT are classified into three types as type 1 and 2 are unstable and type 3 is stable. All studies were performed by one radiologist.

### Magnetic resonance imaging and dynamic contrast enhanced angiography

All MRI examinations were performed on a 1.5-T unit (Magnetom Avanto, Siemens Medical Solutions, USA). The protocol includes:

1. Scout images for subsequent planning of study in coronary and axial planes with MIP (maximum intensity projection) reconstruction in the sagittal and coronal planes.
2. Isometric T1 based measurement in coronary plane with fat suppression for detailed imaging and localization of plaques and the subsequent planning of axial images with high resolution in the stenotic area.
3. T1 based fat suppression images and high resolution in the axial plane of the level of the plaque - the presence of a lipid core, hemorrhage, calcification, fibrosis.
4. T2-based ECG-triggered images with fat suppression in the axial plane of additional tissue characterization of plaque in T2 measurement - the presence of necrosis, calcification, fibrosis.
5. Proton Dencity (PD) based ECG-triggered images with fat suppression in the axial plane of additional tissue characterization.
6. Dynamic contrast enhanced angiography in coronary plane. Contrast enhancement is achieved by intravenous injection of 0.2 ml/kg Gadopentetate Dimeglumine (Magnevist, Bayer Schering Pharma AG, Germany).
7. Postcontrast T1 images with fat suppression and high resolution in the axial plane (with localization identical to the postcontrast ones). We compared the pre- and postcontrast images and we analyzed the gain in the plaque and vessel wall. We assessed the presence, thickness, and integrity of the fibrous capsule.

The degree of carotid stenosis was evaluated by NASCET criteria. The classification of the lesions was based on AHA classification of coronary atherosclerotic plaques modified for MRI by Cai et al.: type I-II - almost normal thickness without calcification; type III - diffuse intimal thickening or small eccentric plaque without calcification; type IV-V - plaque with lipid and/or necrotic core, surrounded by fibrous tissue with possible calcification; type VI - complicated plaque with a possible defect in the surface, hemorrhage or thrombus; type VII - calcified plaque; type VIII - fibrous plaque without lipid core with a possible slight calcification [28]. All studies were performed by one radiologist.

### Histology

Plaques were removed intraoperatively “en blok” without fragmentation and were fixed in 10% formalin solution. We took 2 to 5 cross-cut material of each plaque in 7-8 mm distance which indicated the most severe atherosclerotic permeates. We made histological preparation slices with thickness of 4 μ and stained with hematoxylin-eosin, Perls (hemosiderin), Van Gieson (collagen/fibrous tissue) and Congo red (fats). Large components such as lipid core, fibrous tissue and calcification are detected and determine the percentage content of the plaque area. The thickness of the fibrous capsule was measured, the presence of old or recent bleeding plaque rupture of the fibrous capsule (absence/presence of fibrous capsule regardless of thickness) was noted and also the presence of old or fresh thrombosis and proximity of the lipid core to the arterial lumen. The plaques are classified into two types: soft unstable plaques, that contain predominantly liquid ingredients (a lipid core and/or hemorrhage in the plate with <70% of fibrous tissue and /or calcification) and solid fibrous plaques consisting mainly of fibrous tissue and/or calcifications with <30% of the lipid core and /or hemorrhage into the plaque.

### Statistics

We defined sensitivity and specificity of the three imaging modalities. The software used was PASW Statistics 18, SPSS Inc. and Microsoft Office 2010.

## Results

### Ultrasound

**Figure 1:**
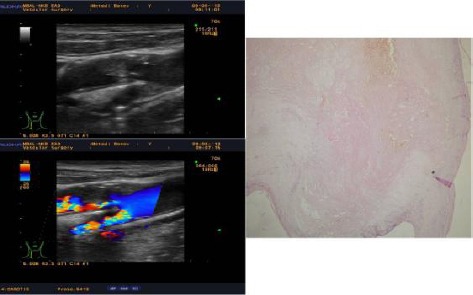
*Unstable soft plaque type I with a deep ulcer causing 95% ICA stenosis in symptomatic 67 years old male with recurrent ipsilateral stroke with B-mode, Color Duplex Sonography and corresponding histological section*.

Ultrasound found 13 type I plaques (Figure 1), 16 type II plaques, 19 Type III plaques and 9 type IV plaques, ie 29 unstable and 28 stable plaques. Histological examination of material from CEA found 31 risk soft plaques and 26 solid fiber calcification. B-scan in grayscale diagnosed with accuracy 29 of 31

histologically proven risk soft plaques and found 28 stable solid plaques in histologically confirmed 26 ones. Carotid duplex sonography showed 93.5% sensitivity and 92.9% specificity for detection of unstable carotid plaque. The presence of carotid plaque ulceration is another independent risk factor for stroke than echogenicity and structure. Duplex sonography found ulceration in 9 plaques, and histological examination revealed a rupture of the fibrous cap in 7 plaques. The sensitivity of CDS in ulcer plaque detection was 77.8% and specificity was 96%.

### MDCT

**Figure 2:**
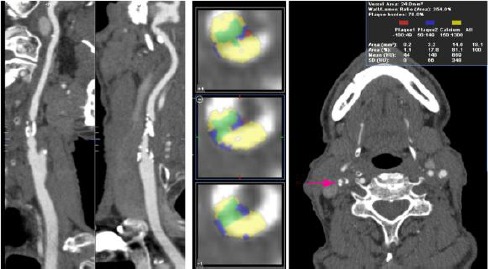
*MDCT of right ICA with unstable plaque with lipid core and ulcer with lumen stenosis of 77%. Left - Multilevel Reconstructed Image. Middle – automated classification computer algorithm derived overlay shows lipid core (blue), blood products (red), calcification (yellow) and connective tissue (green). Right - axial section at the level of the plaque*.

Multidetector computed tomography recognized through comprehensive assessment of carotid plaque morphology (Figure 2) 18 unstable plaques and 11 stable. Histological examination confirmed the presence of 18 unstable plaques and 11 stable ones. The findings of the study of plaque types in MDCT and histology look identical, but this is due to the equal number of false positive and false negative results. With MDCT we recognized three stable plaques as unstable and 3 unstable plaques as stable. We found 83.3% sensitivity and 72.7% specificity with MDCT for detection of unstable carotid plaque.

Classification of carotid plaques only for their density in Hounsfield units gave a different result. The study identified 12 unstable plaques of 18 histologically proven ones and 17 stable of 11 stable from histology. MDCT sensitivity for detection of unstable carotid plaque based solely on density is 61.1% and the specificity is 88%. This result showed a significant divergence in terms of sensitivity to the complex assessment of carotid plaque.

MDCT recognized 8 of 11 histologically proven stable calcium plaques, which shows 72.7% sensitivity and 83.3% specificity for detecting calcium plaques. This result is in the lower limit reported in the literature and is probably due to plaque decalcification during histological examination.

The study showed 19 plaques without ulceration and 10 with histologically confirmed ulcer in 18 plaques with an intact surface and 11 with rupture of the fibrous capsule. The MDCT detection of ulcer in carotid plaque has 90.9% sensitivity and 94.7% specificity.

### MRI

**Figure 3:**
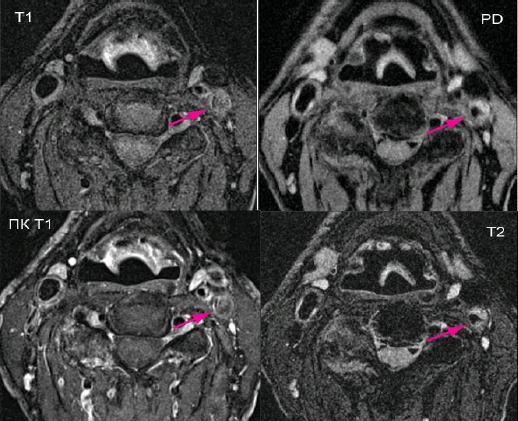
*MRI of unstable carotid plaque in 67 years old symptomatic male, with ulceration (type VIa), occluding 95% of the lumen of the left ICA. It consists of a large lipid core, plaque hemorrhage and a thin fibrous cap (enhanced on postcontrast T1), in which a small area is interrupted (ulceration)*.

According to the AHA classification for MRI in this study we found 22 unstable plaques (type IV-V and VI) and 8 stable (type VII and VIII). It is noteworthy the high incidence of unstable plaques in patients studied with MRI (Figure 3). The ratio unstable to stable plaques is almost 3: 1. Histological examination revealed 21 risk unstable plaques and 9 stable plaques. Magnetic resonance imaging recognized all unstable plaques (21 of 21) and also recognized one stable plaque as unstable. The sensitivity of MRI was 100% and specificity - 88.8% for the detection of unstable carotid plaque. Our results are excellent and suggest that the MRI is the most accurate non-invasive imaging for assessment of carotid plaque morphology.

Magnetic resonance imaging recognized presence of necrotic lipid core in 13 plaques. Histology found 14 plaques with lipid content > 30% of their surface. The sensitivity of this test for the detection of that component is 92.8% and the specificity is 94.1%. MRI scans revealed plaque hemorrhage in 12 of 13 histologically proven plaques hemorrhage. The sensitivity of MRI for detection of hemorrhage was 92.3% and specificity was 94.4%. Magnetic resonance imaging revealed ulceration in 7 carotid plaques. Histological examination found 9 plaques with ruptured fibrous cap. The sensitivity of MRI for detecting plaque ulcers was 77.8% and the specificity was 91.3%.

Summary results for the diagnostic value of the three imaging modalities are presented in Table 1.

**Table 1:** Diagnostic value of imaging modalities.

Study	Sensitivity	Specificity
CDS	94%	93%

CDS - U	78%	96%

MDCT	83%	73%

MDCT - U	91%	95%

MDCT - C	73%	83%

MRI	100%	89%

MRI - L	93%	94%

MRI - H	92%	94%

MRI - U	78%	91%

U- ulcer, L – lipids, C – calcium, H – hemorrhage.

## Discussion

Major criteria as an indication for surgery according to multi-center studies are: carotid stenosis >70%; symptomatic patients with stenoses >50% and plaque hemorrhage or irregular surface and symptomatic patients with ulcerated plaques [7]. Other factors, beside percent of stenosis, are important for assessing whether carotid lesion will remain without clinical manifestations. Unstable plaques are prone to rupture and distal embolization of atheromatous material. Ulcerated plaques are prone to superimpose thrombosis which may lead to a significant increase of the stenosis or thrombosis. Fresh thrombus on ulceration may embolize (part of it or the whole one) and may lead to severe cerebrovascular accident. The presence of liquid components in the plaque can play an important role in deciding the kind of treatment and in determining the treatment plan for patients with carotid stenosis. Solid hard plaques can be safely treated with CEA or CAS, but in the case of CAS, taken in a patient with high-risk soft plaque it is possible the fibrous capsule to rupture during the procedure, which can lead to leakage of the viscous fluid from the plaque. It can pass through distal protection device and cause severe cerebrovascular embolism. In this case, the treatment method of choice is CEA [17, 19, 25].

The purpose of our study was to determine the diagnostic accuracy (sensitivity and specificity) of CDS, MDCT and MRI for detection of unstable carotid plaque compared to histological examination.

Reiter et al. demonstrate sensitivity of CDS for detection of plaque ulceration 100% and specificity 93%. He detects unstable plaques with sensitivity 75% and specificity 88%. He compares the image of the plaque obtained with B-mode modality and the program B-Flow Imaging and does not prove the advantages of the second method over the first one [26].

Ten Kate et al. analyzed eight CDS studies, investigating irregularities in luminal surface of the plaque, and 6 of them - also the presence of ulcers. Ultrasound has a relatively low sensitivity (60%) for the detection of ulcers, while the surface roughness is more accurate (sensitivity 97%, specificity 81%) [25, 27, 28]. The European Carotid Plaque Study Group uses B-scan for detection of ulcerated plaque with sensitivity 47% and specificity 63%. Five studies showed different results with a small improvement in average sensitivity (60%, from 38% to 94%) and specificity (74%, from 33% to 92%). Two studies compared the surface irregularities with plaque composition and prove that their presence in the Ultrasound image predict hemorrhage within the plaque with sensitivity 81% and specificity 85%, while the opposite, heterogeneity and calcification in B-scan are bad predictors for the presence of ulcers [20, 29, 30].

Saba et al. compared directly MDCT with CDS and they conclude that detecting plaque ulceration with CT is more accurate than CDS (sensitivity 93.8% against 37.5% and specificity 98.6% against 91.5%) [25, 31]. Single-slice CT has a sensitivity of 60% and specificity 71% for the detection of ulcer, but MDCT has average sensitivity of 87% (50-94%) and the specificity is 98% on average (89-99%) [29, 32].

Ajduk et al. published direct comparison of MDCT and CDS for detecting plaque hemorrhage, demonstrating a high diagnostic reliability of the first over the second method (sensitivity 100% against 78.2% and specificity 70.4% against 59.2%) [13]. Wintermark et al. shows that only some of characteristics of carotid wall with MDCT were significantly associated with carotid stroke patients. He recognizes the histological structure of the plaque with MDCT in 72.6% of the cases [33-38].

Ten Kate et al. analyzed 18 studies with MRI. Five of them compared the findings of MRI to histology and they find strong coincidence among them for evaluation the wall of the carotid artery. Fragile fibrous cap are detected with high sensitivity (81%) and specificity (90%). Ulcers were captured with a sensitivity of 100% and specificity of 80%. Ten trials have investigated the components of the plaque and the MRI use showed a tight correlation with histology. The lipid core is detected with medium sensitivity of 94% (91-98%) and a specificity of 79% (65-100%). MRI scans can detect as the age of the hemorrhage in the plaque as its location with sensitivity of 88% and specificity of 98%. Combined finding of lipid nucleus and hemorrhage in the plaque was detected with an average sensitivity of 90% (85-94%) and a specificity of 95% (92-100%). The potential of MRI to detect calcifications is questionable due to the lack of water in them. In contrast to this assumption, there is a high coincidence and calcifications were detected with an average sensitivity of 77% (76-80%) and a specificity of 88% (86-94%) [38].

Recent comparative study of Watanabe et al. for MRI and CDS demonstrated sensitivity and specificity for MRI 96% and 93% vs. 75% and 63% for CDS in detecting unstable carotid plaques [39-41]. This is a huge improvement over Yoshida et al. (sensitivity 79% and specificity 84%). Cai et al found high sensitivity (80-84%) and specificity (90-98%) of MRI for detection of plaque type III-VII [28].

Weakness of our work is the little number of patients studied with MDCT and MRI. The reason is the high costs of these imaging techniques in Bulgaria.

Our results show the highest sensitivity and specificity of MRI for detection of unstable carotid plaques and their histological components but it is expensive, takes a long time and is difficult to access. Ultrasound assessment of plaque morphology and the presence of ulceration have very high sensitivity and specificity. We confirmed that CDS is accessible, reliable, cheap and very useful tool for the final diagnosis, grading the risk of stroke and determining the treatment plan for patients with carotid stenosis. Limitations of this method are known but only few cases require additional imaging. MDCT determines exactly the percent of stenosis, finds perfectly calcification and ulceration, and shows good correlation with histology for large lipid core and major hemorrhage. There is overlap between the densities of lipid necrotic core, fibrous tissue and hemorrhage, which seriously limits the ability of the methodology for determining the morphology of unstable carotid plaque. The method is limited by radiation exposure and contrast. The three noninvasive imaging methods are complementary, avoiding weaknesses, for optimal assessment of carotid plaque morphology (Table 2).

**Table 2:** Characteristics of noninvasive imaging modalities.

Imaging modality	Costs (€)	Time (min)	Advantages	Disadvantages
US	40	20	- Accessible - Safe - Repeatable with possibility for tracking of stenosis and therapy effect - Fast – real-time without need for post procedure processing - Screening and diagnostics - Anatomy and function - Cheap	- Difficult - in “hostile” neck - When high bifurcation – uninformative - Calcium plaques with acoustic shadowing - Difficult assessment of heterogeneous plaque vulnerability - Homogeneous plaque– fibrosis or lipids? - Subjectivism
MDCT	150	55	- Accessible - In one image – percent stenosis and plaque morphology - Calcifications - Plaque ulceration	- Toxic contrasts - Radiation exposure - Poor soft tissue differentiation - Subjectivism
MRI	220	70	- Measures the thickness of the fibrous cap - Discovers: rupture of the fibrous cap; plaque hemorrhage; lipid-necrotic core; calcifications; inflammation; neoangiogenesis.	- Poor image quality - Motion artifacts - Toxic contrasts - Long investigating time - Hard accessible - Expensive - 5% of patients are unsuitable

US – ultrasound, MDCT - Multidetector computed tomography, MRI - Magnetic resonance imaging.

It is impossible for us to study all patients with the three imaging techniques because of the high costs, risks and difficult access to MDCT and MRI, which is very important for symptomatic patients. These patients benefit the most from CEA in the first two weeks after TIA and stroke. The first step is screening with CDS, which gives us information about whether to direct the patient directly for CEA or to deepen the study of carotid arteries with more specialized and expensive imaging technique (MDCT or MRI).

**Figure 4:**
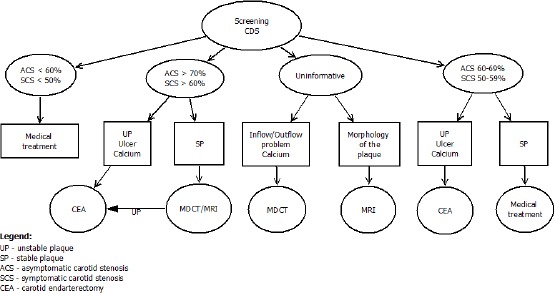
*Diagnostic algoritm for extracranial stenosis*.

The results of our study and literary data give us a reason to develop an algorithm for the diagnosis of extracranial carotid stenoses, including new methods for assessing the composition and morphology of atherosclerotic plaque (Figure 4).
